# Potential involvement of *Helicobacter pylori* from oral specimens in overweight body-mass index

**DOI:** 10.1038/s41598-019-41166-5

**Published:** 2019-03-19

**Authors:** Masakazu Hamada, Ryota Nomura, Yuko Ogaya, Saaya Matayoshi, Tamami Kadota, Yumiko Morita, Narikazu Uzawa, Kazuhiko Nakano

**Affiliations:** 1 Department of Oral and Maxillofacial Surgery II, Osaka University Graduate School of Dentistry, Suita, Osaka, Japan; 2 Department of Pediatric Dentistry, Osaka University Graduate School of Dentistry, Suita, Osaka, Japan

## Abstract

The bacterium *Helicobacter pylori* was originally classified in the *Campylobacter* genus, which contains major periodontopathic bacterial species, and *H*. *pylori* DNA has been found in the oral cavity. Although many studies show an association between the presence of periodontal bacteria and an overweight body-mass index (BMI; >25 kg/m^2^), the relationship between body weight and the presence of *H*. *pylori* in the oral cavity has not been demonstrated. Herein, we analysed the relationship between *H*. *pylori* in the oral cavity and systemic conditions, including the overweight BMI. Saliva specimens and extracted teeth were obtained from 87 subjects; the distribution of *H*. *pylori* among these specimens was analysed with the polymerase chain reaction. Subjects with an overweight BMI exhibited significantly higher detection rates of *H*. *pylori* in saliva, compared with non-overweight subjects (BMI <25 kg/m^2^) (*P* < 0.05). A clinical history of digestive diseases was not associated with the presence of *H*. *pylori* in overweight subjects, whereas subjects with both severe dental caries and an overweight BMI showed a higher detection rate of *H*. *pylori* in saliva specimens, compared with other groups. These results suggest that the detection of *H*. *pylori* in the oral cavity could be associated with the overweight BMI, which was predominant among subjects with severe dental caries.

## Introduction

*Helicobacter pylori* is a Gram-negative microaerophilic bacterium which is mainly isolated from the stomach and duodenum^1^. *H*. *pylori* was previously classified in the *Campylobacter* genus, which also contains several major periodontal bacterial species; initial infection by the bacterium is considered to occur mainly in childhood, before the age of 5 years, via the oral cavity^1^. Although some individuals harbouring *H*. *pylori* exhibit no symptoms, chronic infection by the bacterium increases the risk of gastric diseases, such as peptic ulcers or cancer^2^.

The existence of *H*. *pylori* in gastric tissue is diagnosed by endoscopic biopsy, serum antibody, urea breath test, and/or stool antigen test^3^; polymerase chain reaction (PCR) methods are widely used for the detection of *H*. *pylori* in the oral cavity^4^. According to previous studies that have shown *H*. *pylori* detection in oral specimens, such as dental plaque and saliva, by using PCR methods, the oral cavity may be a potential reservoir and reinfection source of *H*. *pylori*^5^. The prevalence of *H*. *pylori* in saliva specimens obtained from Japanese subjects was estimated to be 6.4%, using the PCR method^6^. Although a large number of PCR methods are available, the detection of *H*. *pylori* from among approximately 700 oral bacterial species—with high specificity and sensitivity—is quite difficult^7,8^. Thus, we recently developed a novel nested PCR procedure by using reliable primer sets, which were designed based on genome sequences from approximately 50 *H*. *pylori* strains^8,9^.

Overweight and obese body-mass indexes (BMIs) constitute a global problem, especially in developed countries, as one of the most important risk factors for metabolic syndrome and various diseases^10,11^. Many studies have shown a positive association between the presence of *H*. *pylori* in gastric tissue and the onset of metabolic syndrome^12–14^. Additionally, it is widely known that periodontal bacterial species are closely associated with the presence of systemic diseases that involve weight change, such as diabetes, obesity, and non-alcoholic fatty liver disease^15–17^. Furthermore, recent studies have shown that infection with specific cariogenic bacteria is associated with increased body weight^18,19^. However, the relationship between *H*. *pylori* colonization in the oral cavity and increased body weight remains unknown^20^. In the present study, we investigated the relationship between *H*. *pylori* colonization in the oral cavity and a variety of clinical factors, with particular focus on overweight BMI.

## Results

### Clinical characteristics of *H*. *pylori*-positive and *H*. *pylori*-negative subjects

A total of 87 subjects were divided into two groups based on the absence (*H*. *pylori*-negative) (n = 71; 81.6%) or presence (*H*. *pylori*-positive) (n = 16; 18.4%) of *H*. *pylori* in the oral cavity. *H*. *pylori*-positive subjects were further stratified into those who exhibited *H*. *pylori* in saliva (n = 8; 9.2%) and those who exhibited *H*. *pylori* in extracted teeth (n = 13; 14.9%) (Table 1); five subjects (5.7%) exhibited *H*. *pylori* in both saliva and extracted teeth. Among these specimens, six and one *H*. *pylori*-positive saliva specimens showed 1.0 × 10^2^–1.0 × 10^3^ CFU and 1.0 × 10^3^–1.0 × 10^4^ CFU of *H*. *pylori*, respectively. Only one *H*. *pylori*-positive saliva specimens included 1.0 × 10^4^–1.0 × 10^5^ CFU of *H*. *pylori*. In contrast, eight and five *H*. *pylori*-positive extracted teeth showed 1.0 × 10^2^–1.0 × 10^3^ CFU and 1.0 × 10^3^–1.0 × 10^4^ CFU of *H*. *pylori*, respectively. Mean age and height were similar among all groups, whereas the body weights and BMIs were higher in subjects with *H*. *pylori* in saliva, compared with all other groups. In addition, a greater proportion of subjects with *H*. *pylori* in saliva and/or extracted teeth were male, had a history of systemic diseases, and/or had a history of gastrointestinal diseases, compared with subjects without *H*. *pylori*. Furthermore, medical history and previous eradication of *H*. *pylori* from gastric tissue did not correlate with detection of *H*. *pylori* in the oral cavity.

**Table 1:** Comparison of clinical characteristics between *H. pylori*-positive and *H*. *pylori*-negative groups.

Clinical characteristics	*H*. *pylori*-negative (n = 71)	*H*. *pylori*-positive (n=16)	
		Saliva (n = 8)	Teeth (n = 13)
Age (years; mean ± SD)	45.6 ± 20.7	48.4 ± 15.3	50.5 ± 20.1
Sex (Male (%))	37 (52.1)	5 (62.5)	8 (61.5)
Height (cm; mean ± SD)	163.5 ± 8.4	164.6 ± 8.9	163.8 ± 7.4
Body weight (kg; mean ± SD)	58.5 ± 10.5	66.3 ± 8.5	60.6 ± 11.6
BMI (kg/m^2^; mean ± SD)	21.8 ± 2.7	24.5 ± 3.0	22.5 ± 3.5
History of systemic disease (%)	30 (42.3)	5 (62.5)	9 (69.2)
History of gastrointestinal disease (%)	12 (16.9)	2 (25.0)	4 (30.8)
Medical history of *H*. *pylori* infection in gastric tissue (%)	7 (9.9)	0 (0)	1 (7.7)
Previous eradication of *H*. *pylori* in gastric tissue (%)	3 (4.2)	0 (0)	1 (7.7)

In regard to dental caries status, the number of decayed teeth was higher in *H*. *pylori*-negative subjects, compared with *H*. *pylori*-positive subjects. In contrast, the numbers of missing teeth, filled teeth, and decayed, missing, or filled teeth (DMFT, combination of the three previous values) were higher in *H*. *pylori*-positive subjects (Table 2). The mean depth of the periodontal pocket around the extracted teeth (prior to extraction) was greater in subjects with *H*. *pylori* in saliva and in *H*. *pylori*-negative subjects, compared with subjects with *H*. *pylori* in extracted teeth.

**Table 2:** Dental caries status and periodontal pocket characteristics compared between *H. pylori*-positive and *H*. *pylori*-negative groups.

Clinical characteristics	*H*. *pylori*-negative (n = 71)	*H*. *pylori*-positive (n=16)	
		Saliva (n = 8)	Teeth (n = 13)
DT index (mean ± SD)	1.8 ± 2.5	1.0 ± 0.8	1.4 ± 2.1
MT index (mean ± SD)	2.8 ± 5.1	3.4 ± 4.4	4.5 ± 5.5
FT index (mean ± SD)	7.7 ± 5.4	10.6 ± 4.9	9.7 ± 5.2
DMFT index (mean ± SD)	12.3 ± 8.3	15.0 ± 6.9	15.5 ± 8.0
Periodontal pocket (mean ± SD)	4.5 ± 3.3	4.8 ± 2.3	3.8 ± 1.5

### Distribution of *H*. *pylori* in subjects with or without overweight BMI

**Figure 1:**
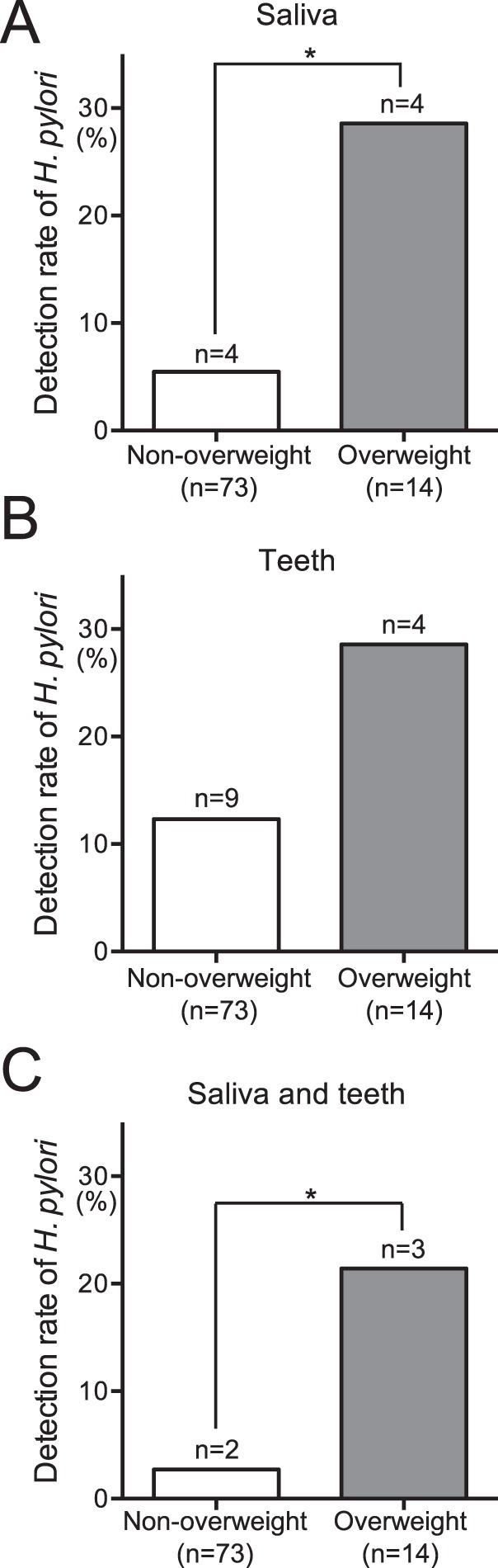
Detection of *H. pylori* in oral specimens obtained from overweight and non-overweight subjects. Saliva (**A**), teeth (**B**) and saliva and teeth combined (**C**) were analysed. Significant differences were determined by using ANOVA with Bonferroni correction. *P < 0.05 between two groups.

BMI >25 kg/m^2^ was defined as overweight in the present study^21^. Subjects were classified into a non-overweight group (BMI <25 kg/m^2^; n = 74) and an overweight group (BMI >25 kg/m^2^; n = 13). In terms of *H*. *pylori* detection from saliva, the overweight group exhibited a significantly higher rate of detection than the non-overweight group (*P* < 0.05; Fig. 1A). A similar trend was observed regarding the rate of *H*. *pylori* detection in extracted teeth, although the statistical evidence was weak (Fig. 1B). In addition, a significant difference was observed in rates of *H*. *pylori* detection between extracted teeth and saliva (*P* < 0.05; Fig. 1C).

### Distribution of *H*. *pylori* in relation to other clinical factors

**Figure 2:**
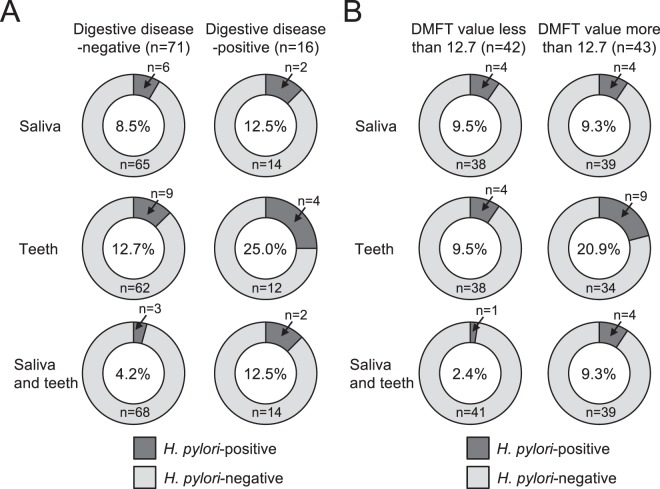
Comparison of the rates of *H. pylori* detection in oral specimens obtained from subjects with different clinical statuses. (**A**) The rates of *H*. *pylori* detection in subjects with or without digestive disease. (**B**) The rates of *H*. *pylori* detection in subjects with decayed, missing, or filled teeth (DMFT) values of <12.7 or >12.7. Rates of *H*. *pylori* detection are shown in centres of pie graphs.

Subjects were classified into two groups: those with digestive diseases in their clinical history (n = 16) and those without (n = 71). The group with a history of digestive diseases showed higher rates of *H*. *pylori* detection, compared with the group without a history of digestive diseases (Fig. 2A).

Next, all subjects were divided into two groups based on the severity of dental caries status. The average DMFT for all subjects was 12.7; thus, we stratified subjects into a group with DMFT < 12.7 (low DMFT, n = 42) and a group with DMFT > 12.7 (high DMFT, n = 43) (Fig. 2B). Higher detection rates of *H*. *pylori* were observed in the high DMFT group, compared with the low DMFT group. We further stratified *H*. *pylori* detection rates based on periodontal pocket depths of the extracted teeth; subjects were divided into two groups as follows: subjects with periodontal pockets <4 mm (n = 37) and subjects with periodontal pockets >4 mm (n = 34) (Supplemental Fig. 1). Greater periodontal pocket depth did not correlate with a higher rate of *H*. *pylori* detection.

### Distribution of *H*. *pylori* according to BMI and clinical history of digestive disease

**Figure 3:**
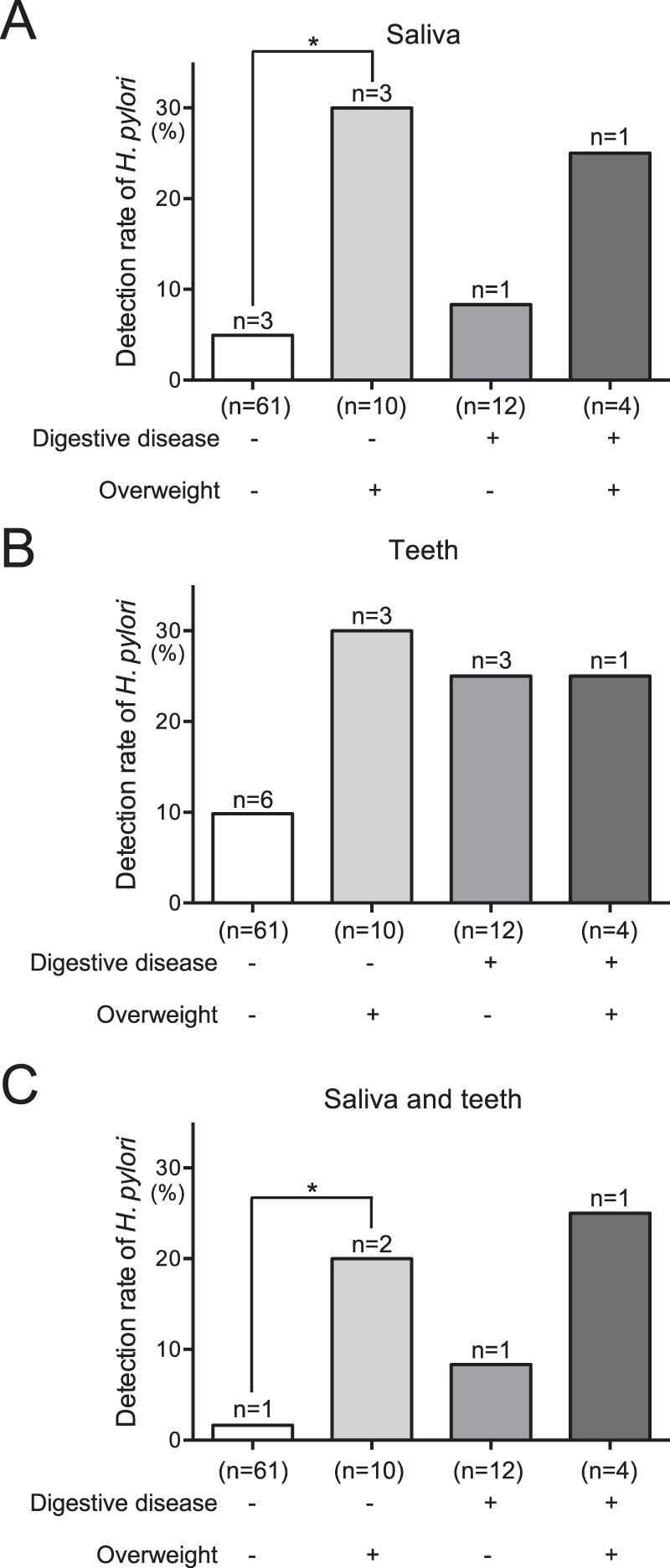
Detection of *H. pylori* in oral specimens obtained from overweight subjects, with or without digestive diseases. Saliva (**A**), teeth (**B**) and saliva and teeth combined (**C**) were analysed. Significant differences were determined by using ANOVA with Bonferroni correction. *P < 0.05 between two groups.

Subjects were divided into four groups according to BMI and clinical history of digestive diseases, as follows: digestive disease-negative and non-overweight BMI (n = 61), digestive disease-negative and overweight BMI (n = 10), digestive disease-positive and non-overweight BMI (n = 12), and digestive disease-positive and overweight BMI (n = 4). Among all oral specimens, there were no significant differences in rates of *H*. *pylori* detection between overweight subjects with or without digestive disease (Fig. 3A–C). Overweight subjects without digestive disease showed significantly higher rates of *H*. *pylori* detection in saliva alone and in saliva and extracted teeth combined, compared with non-overweight subjects without digestive disease (*P* < 0.05).

### Distribution of *H*. *pylori* according to BMI and dental caries status

**Figure 4:**
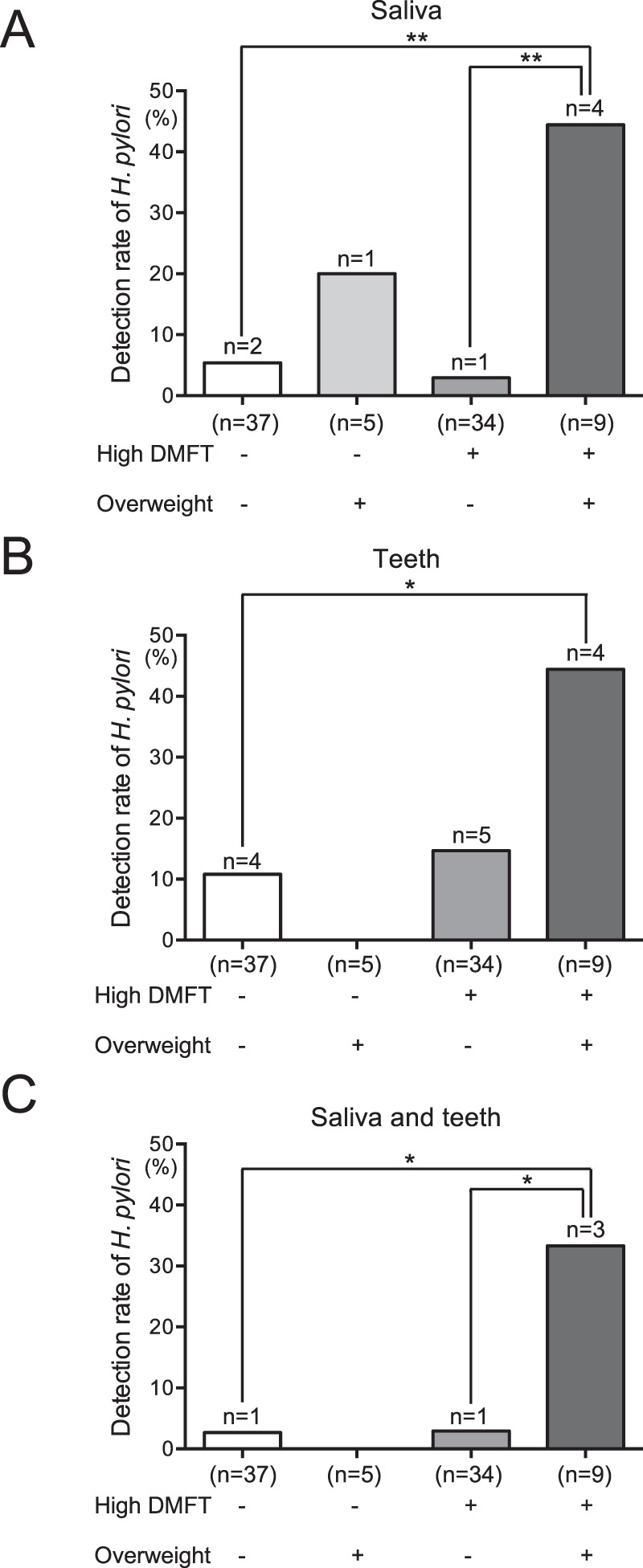
Detection of *H. pylori* in oral specimens obtained from overweight subjects, with or without high numbers of decayed, missing, or filled teeth (DMFT). Saliva (**A**), teeth (**B**) and saliva and teeth combined (**C**) were analysed. Significant differences were determined by using ANOVA with Bonferroni correction. *P < 0.05 and **P < 0.01 between two groups.

Subjects were divided into four groups according to BMI and number of DMFT, as follows: DMFT < 12.7 and non-overweight BMI (n = 37), DMFT < 12.7 and overweight BMI (n = 5), DMFT > 12.7 and non-overweight BMI (n = 34), and DMFT > 12.7 and overweight BMI (n = 9). Overweight subjects with high numbers of DMFT demonstrated the highest rates of *H*. *pylori* detection in all oral specimens (Fig. 4A–C); this rate was significantly higher than in non-overweight subjects with low numbers of DMFT (*P* < 0.05). Overweight subjects with high numbers of DMFT showed significantly higher rates of *H*. *pylori* detection in saliva alone and in saliva and extracted teeth combined, compared with non-overweight subjects with high numbers of DMFT (*P* < 0.05).

## Discussion

The oral cavity has been recognized as a possible reservoir for *H*. *pylori*, and therefore might serve as a source of bacteria for gastric infection or transmission to others^1^. Prior epidemiological studies have reported an association between the presence of *H*. *pylori* in gastric tissues and the occurrence of digestive diseases and metabolic syndromes^2,22^. In addition, the effects of *H*. *pylori* from the oral cavity on the pathogenesis of digestive diseases have also been investigated^23^. However, there have been no studies regarding the relationship between *H*. *pylori* in the oral cavity and body weight. Notably, altered body weight has a relationship with various systemic diseases, especially digestive diseases and metabolic syndromes. To the best of our knowledge, this is the first study to demonstrate that the presence of *H*. *pylori* in the oral cavity is associated with overweight BMI.

Recently, oral bacteria were reported to be associated with various systemic diseases^24^. Moreover, significant associations between the presence of specific periodontal bacterial species and the occurrence of systemic diseases (e.g., diabetes, obesity, cardiovascular disease, and non-alcoholic fatty liver disease) have been reported^15–17,25^. *H*. *pylori* was originally classified in the *Campylobacter* genus, which contains multiple major periodontopathic bacteria^2^. *Campylobacter rectus*, a common *Campylobacter* species in the oral cavity, is associated with systemic diseases, such as low birth weight^26^; furthermore, *C*. *rectus* DNA has been detected within atheromatous plaque^27,28^. Therefore, it is reasonable to speculate that the presence of *H*. *pylori* in the oral cavity may be associated with the occurrence of overweight BMI, thereby acting as an exacerbating factor for subsequent systemic diseases.

Overweight and obese BMI (i.e., increased BMI) is considered a major risk factor for periodontal diseases, and the reverse association has also been reported^29^. In obese subjects, inflammatory cytokines are produced by adipose cells and secreted into the bloodstream^30^, resulting in immunodeficiency against infectious periodontal bacteria and subsequent destruction of periodontal tissue^31^. Moreover, bacterial profiles in saliva were altered in obese subjects, compared with those in healthy individuals^32^; the levels of some periodontopathic bacteria were significantly higher in obese subjects than in healthy individuals^33^. Although blood analyses were not performed in the present study, inflammatory cytokines secreted from adipose cells may also affect the detection of *H*. *pylori* in overweight subjects.

There were significant differences between overweight (28.6%) and non-overweight subjects (5.5%) with respect to the rates of *H*. *pylori* detection in saliva. The detection rate of *H*. *pylori* in overweight patients in the present study was also higher than the detection rate of *H*. *pylori* in saliva obtained from Japanese subjects without gastric diseases (6.4%; 21 of 326 subjects)^6^. However, no significant differences were observed in the rates of *H*. *pylori* detection in extracted teeth. These results indicate that the presence of *H*. *pylori* in saliva is related to the occurrence of overweight BMI.

In the present study, *H*. *pylori* ranges between 1.0 × 10^2^–1.0 × 10^5^ CFU/mL were observed in *H*. *pylori*-positive saliva specimens from overweight subjects. Daily production of saliva in a healthy person is approximately 0.5–1.5 L^34^. Therefore, approximately 5 × 10^4^–1.5 × 10^8^ CFUs of *H*. *pylori* may be present in secreted saliva each day. These bacteria could be swallowed and reach gastric tissue, thereby contributing to the occurrence of overweight BMI. Indeed, periodontal bacteria that were swallowed in saliva have been reported to induce systemic diseases, and some of these diseases exhibit altered gut microbial composition associated with obesity^35^. Although we only focused on *H*. *pylori* in the oral cavity in the present study, comparison of quantification data between *H*. *pylori* in the oral cavity and that in gastric tissue may be important for determining how many *H*. *pylori* in the oral cavity are related to the colonization of *H*. *pylori* in gastric tissue.

In our previous study, we sampled oral specimens from eight *H*. *pylori*-positive specimens in the first sampling^9^; seven of these eight specimens were *H*. *pylori*-positive in the second sampling. Furthermore, among twelve *H*. *pylori*-negative specimens in the first sampling, only one showed positive results in the second sampling. Based on these results, we concluded that *H*. *pylori* was not transiently present, but that it colonized the oral cavity for an extended period. However, in that study, the intervals between first and second samplings were short (approximately 1–2 weeks) and the sample sizes were quite low; moreover, that study did not focus on overweight subjects. Thus, further studies are needed to confirm whether *H*. *pylori* is transient, using oral samples taken from overweight subjects at two or three separate times.

Periodontal inflammation induced by periodontal bacteria in subjects with periodontitis causes inflammatory responses in systemic diseases^36^. In addition, higher levels of inflammatory cytokines have been detected in the periodontal pockets of subjects with periodontitis and obesity, compared with those of non-obese subjects^37^. In the present study, there was no correlation between periodontal pocket depth and the rates of *H*. *pylori* detection. Our results suggest that daily swallowing of *H*. *pylori* in saliva may be a major cause of overweight BMI, and that this BMI may not be related to the production of inflammatory cytokines associated with the presence of *H*. *pylori* in severe periodontitis.

When we analysed subjects without a clinical history of digestive diseases, the rate of *H*. *pylori* detection in overweight subjects was significantly higher than in non-overweight subjects. This indicates that a clinical history of digestive diseases may not be involved in the relationship between the presence of *H*. *pylori* in the oral cavity and overweight BMI. Thus, in some cases, oral *H*. *pylori* may be non-specifically involved in overweight BMI and related systemic diseases simply through its role as an oral bacterial species, in contrast to its role in the onset of digestive diseases induced in gastric tissue.

Dental caries may be a risk factor for infection by *H*. *pylori* in the oral cavity^8,38^, consistent with the present results. Among subjects with high numbers of DMFT, overweight subjects showed significantly higher rates of *H*. *pylori* detection, compared with non-overweight subjects. Although it remains unclear why dental caries and overweight BMI are risk factors for the presence of *H*. *pylori* in the oral cavity, daily dietary habits may be involved: excessive food intake, especially in terms of sugar, is associated with increased risks for dental caries and overweight BMI. Thus, other factors, such as dietary habits, should be analysed in future studies.

There are some limitations in the present study. First, this study enrolled a small number of subjects and none were defined as obese (BMI >30). Therefore, we focused on the detection of oral *H*. *pylori* in overweight subjects (BMI >25). In addition, the relationship between *H*. *pylori* infection in gastric tissue and the presence of *H*. *pylori* in the oral cavity was ambiguous, as few subjects reported a medical history of *H*. *pylori* infection in gastric tissue (n = 8) or previous eradication of *H*. *pylori* from gastric tissue (n = 4). Further studies should be performed with a larger number of samples; moreover, it may be more relevant to construct a collaborative study involving both dentists and gastroenterologists. Second, we revealed that detection of *H*. *pylori* in the oral cavity was associated with a high frequency of overweight BMI. Unfortunately, the present study did not demonstrate a mechanism by which oral *H*. *pylori* might contribute to the development of overweight BMI. It is possible that *H*. *pylori* in the oral cavity may induce overweight BMI in a similar manner to canonical periodontal bacteria. Therefore, we should perform confirmatory *in vitro* and *in vivo* experiments, similar to those previously used to show the mechanism by which obesity was induced by periodontal bacterial species^18,39^. Third, since this was a retrospective study with a limited number of subjects, prospective studies involving larger numbers of subjects should be performed to confirm our hypotheses.

In summary, detection of *H*. *pylori* in the oral cavity was associated with overweight BMI, especially in subjects with a severe dental caries status.

## Methods

### Ethics statement

This study was conducted in full adherence to the Declaration of Helsinki. The study protocol was approved by the Ethics Committee of Osaka University Graduate School of Dentistry (approval no. H23-E1-5). Prior to specimen collection, all subjects were informed of the study protocol and provided written informed consent.

### Subjects and clinical data

Saliva and extracted teeth were obtained from 87 subjects (46 males and 41 females, age range: 20–83 years; median: 40 years; mean age: 45.3 ± 20.2 years) who were referred to the Department of Oral and Maxillofacial Surgery at Osaka University Dental Hospital from March 2016 to June 2017 due to dental problems requiring tooth extraction, such as dental caries and periodontitis. Approximately 1 mL of saliva or extracted teeth in 2.5 mL sterile saline were collected in sterile disposable tubes and used in the following study. The following information was collected from all subjects: height, weight, medical history, prior incidence of *H*. *pylori* infection in gastric tissue, and incidence of previous eradication of *H*. *pylori* from gastric tissue.

### Evaluation of dental caries status

The dental caries status of all subjects was evaluated by a single dentist following visual inspection and findings from panoramic photographs. The clinical dental examination included the number of total teeth, decayed teeth, missing teeth and filled teeth. The numbers of DMFT were calculated as described previously^40^. In addition, probing depth in the deepest periodontal pocket of the extracted teeth was measured by using a round-ended probe before extraction.

### Bacterial strains and growth condition

*H*. *pylori* reference strain J99 (ATCC 700824) was used as a positive control for nested PCR. J99 was cultured on blood agar plates (Becton Dickinson, Franklin Lakes, NJ, USA) at 37 °C for 3 days, as described previously^8^. Colonies were then inoculated in 5 mL tryptic soy broth (Difco Laboratories, Detroit, MI, USA), and incubated at 37 °C for 3–5 days under microaerophilic conditions.

### Molecular biological analysis

Bacterial DNA was extracted from 1 mL saliva and extracted teeth in 2.5 mL sterile saline from each of the 87 subjects and resuspended in 100 μL distilled water, as described previously^8^. Then, nested PCR was performed on the bacterial DNA by using primer sets designed in our previous study^9^. Briefly, first-step PCR was performed with 1 μL bacterial DNA in reactions of 20 μL total volume by using primers *ureA*-aF (5′-ATG AAA CTC ACC CCA AAA GA-3′) and *ureA*-bR (5′-CCG AAA GTT TTT TCT CTG TCA AAG TCT A-3′). Second-step PCR was performed with 1 μL of the first PCR product as a template in reactions of 20 μL total volume by using primers *ureA*-bF (5′-AAA CGC AAA GAA AAA GGC ATT AA-3′) and *ureA*-aR (5′-TTC ACT TCA AAG AAA TGG AAG TGT GA-3′). The first and second-step PCR amplifications were performed by using the *TaKaRa Ex Taq* (Takara Bio. Inc., Otsu, Japan) with the following cycling parameters: an initial denaturation at 95 °C for 4 minutes and then 30 cycles of 95 °C for 30 seconds, 55 °C for 30 seconds, and 72 °C for 30 seconds; the final extension was performed at 72 °C for 7 minutes. All PCR products were checked by electrophoresis in a 1.5% agarose gel. The limit of *H*. *pylori* detection by nested PCR was 1–10 CFU^9^. Therefore, nested PCR could detect *H*. *pylori* at levels of more than 1.0 × 10^2^–1.0 × 10^3^ CFU in 1 mL saliva, or in the saline rinse of an extracted tooth. Based on the information from the previous analysis^9^, we performed nested PCR using serially diluted bacterial DNA, as in our previous studies^9,41^; this enabled determination of the approximate numbers of *H*. *pylori* in oral specimens.

### Statistical analysis

Statistical analyses were performed by using the computational software package GraphPad Prism 6 (GraphPad Software Inc., La Jolla, CA, USA). Intergroup differences in each analysis were determined by using analysis of variance (ANOVA) with Bonferroni correction. A *P* value of < 0.05 was considered to be statistically significant.

## Supplementary information

Supplementary figure 1

## Data Availability

The data are not available for public access because of patient privacy concerns, but are available from the corresponding author on reasonable request.
