# Supplementary material for: Potential involvement of Helicobacter pylori from oral specimens in overweight body-mass index
Source: Sci Rep. 2019 Mar 19;9:4845. doi: 10.1038/s41598-019-41166-5 (PMC6425031; doi:10.1038/s41598-019-41166-5)
Supplement: Supplementary file 1 — Supplementary figure 1 [file 41598_2019_41166_MOESM1_ESM.pdf]

Potential involvement of *Helicobacter pylori* from oral specimens in overweight body-mass index

Masakazu Hamada, Ryota Nomura, Yuko Ogaya, Saaya Matayoshi, Tamami Kadota, Yumiko Morita, Narikazu Uzawa and Kazuhiko Nakano

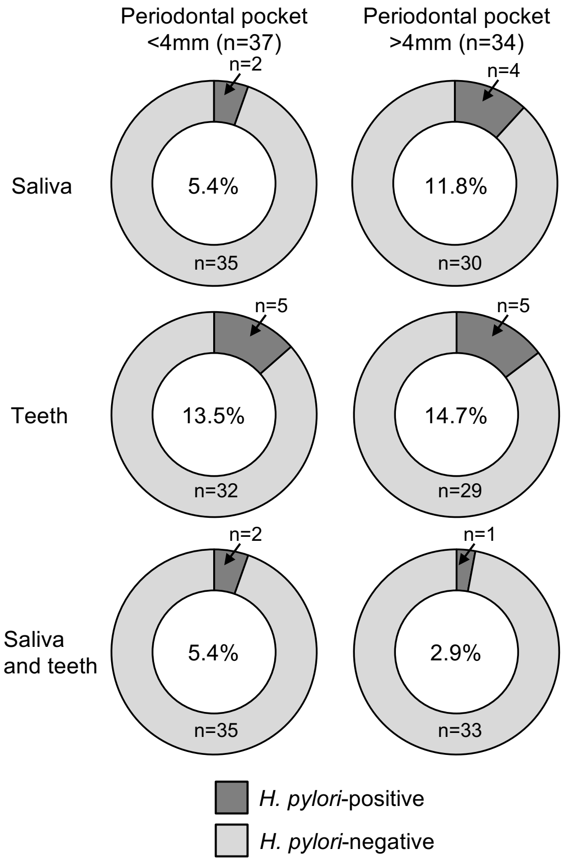

Supplementary Fig. 1. Comparison of the detection rates of *H. pylori* from oral specimens obtained from patients with periodontal pocket <4mm or that >4mm.
